# Diarrhetic Shellfish Poisoning Toxins: Current Insights into Toxicity, Mechanisms, and Ecological Impacts

**DOI:** 10.3390/md24010009

**Published:** 2025-12-23

**Authors:** Hajar Bouda, Rajae El Bourki, Abderrazzak Fattah, Nadia Takati

**Affiliations:** Interdisciplinary Laboratory of Fundamental and Applied Sciences, Higher Normal School, Hassan II University, Casablanca 20000, Morocco; rajaeelbourki@gmail.com (R.E.B.); a.fattah@enscasa.ma (A.F.)

**Keywords:** DSTs, DSP, okadaic acid, dinophysistoxins, harmful algal blooms, marine biotoxins, detection methods, public health, environmental impact

## Abstract

Diarrheic shellfish toxins (DSTs), especially okadaic acid (OA) and its related compounds, are lipophilic marine biotoxins mainly synthesized by dinoflagellates of the genera *Dinophysis* and *Prorocentrum.* These compounds bioaccumulate in filter-feeding shellfish like mussels and clams, posing a considerable public health risk due to their strong gastrointestinal effects when contaminated seafood is consumed. This review offers a thorough overview of the current understanding of OA-group toxins with a focus on the molecular mechanisms of toxicity, including cytoskeletal disruption, apoptosis, inflammation, oxidative stress, and mitochondrial dysfunction. Additionally, their ecological impacts on aquatic organisms and patterns of bioaccumulation are explored. Recent advances in detection methods and regulatory frameworks are discussed, highlighting the necessity for robust monitoring systems to safeguard seafood safety. Enhanced knowledge of the toxicity, distribution, and fate of DSP (diarrheic shellfish poisoning) is essential for improving risk assessment and managing marine biotoxins. Despite methodological advances, gaps remain regarding chronic exposure and species-specific detoxification pathways.

## 1. Introduction

In recent decades, the frequency, geographic distribution, and intensity of harmful algal blooms (HABs) have increased markedly worldwide, raising growing concerns for marine ecosystem health, seafood safety, and coastal economies [1]. HABs, also known as “red tides”, are episodic proliferations of phytoplankton in marine ecosystems able to produce toxic metabolites [2].

**Figure 1:**
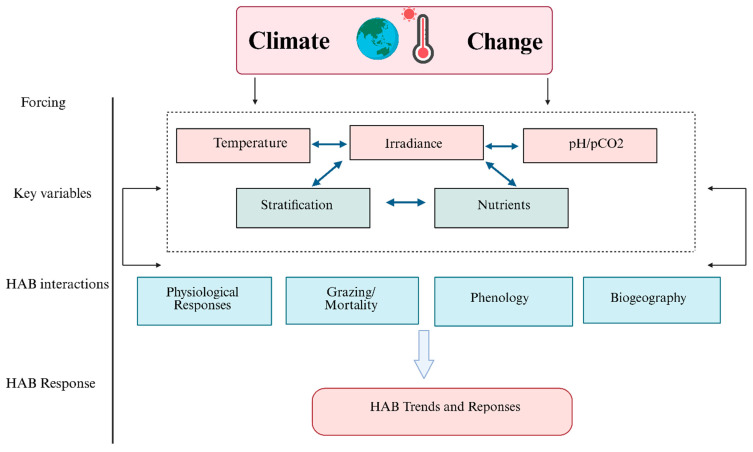
The progression of climate change pressure on key variables and related HAB interactions that will drive HAB responses in the future ocean. Created with Biorender.com.

These naturally occurring toxins are synthesized by unicellular microalgae [3] and bioaccumulate through the aquatic food chain in organisms such as bivalves, xanthid crabs, and pufferfish, potentially posing health risks to humans depending on the amount consumed and considerable economic consequences [4,5]. Anthropogenic activities, including nutrient enrichment from agriculture runoff, intensive coastal exploitation, alterations in water flow dynamics and the introduction of non-native species via ballast waters [6], significantly contribute to the global spread of harmful algal blooms (HABs). The widespread use of detergents, whose consumption has increased markedly over recent decades, further exacerbates this phenomenon [7,8]. Climate change acts as a critical amplifier influencing HAB dynamics through ocean acidification, sea surface warming, altered water column stratification, precipitation-induced nutrient input and changes in light availability [6]. Temperature plays a central role in determining phytoplankton community structure and bloom development, affecting germination [9] motility, photosynthesis, nutrient uptake [10], and ultimately toxin production, as illustrated in Figure 1.

Bivalves are particularly affected by marine toxin contamination, with major social and economic repercussions [11]. Between 2010 and 2015, their global production exceeded 15 million tons annually, generating over USD 23 billion per year, while contamination events cause multimillion-dollar losses worldwide [12]. Marine biotoxins can be classified according to four main criteria: (i) source of isolation, (ii) solubility, (iii) chemical structure, and (iv) toxic effects. Based on these characteristics, several major toxin groups have been identified in marine environments, including paralytic shellfish poisoning (PSP) caused by saxitoxin (STX) and related analogs; tetrodotoxins (TTXs); amnesic shellfish poisoning (ASP) caused by domoic acid; neurotoxic shellfish poisoning (NSP) caused by brevetoxins; diarrheic shellfish poisoning (DSP) caused by okadaic acid; azaspiracid poisoning (AZP) caused by azaspiracids; yessotoxins (YTXs); palyotoxins; ciguatoxins (CTX): and cyclic imines (CIs) [13]

Among these toxin groups, DSP stands out for its widespread occurrence and its implication in numerous contamination events linked to okadaic acid (OA) and its analogs. DSP is primarily associated with okadaic acid (OA) and its derivatives, the dinophysistoxins (DTX-1, DTX-2, and their esterified forms, collectively referred to as DTX-3). Secondarily, DSP may also arise from the ingestion of other filter-feeding organisms that bioaccumulate these toxins.

**Figure 2:**
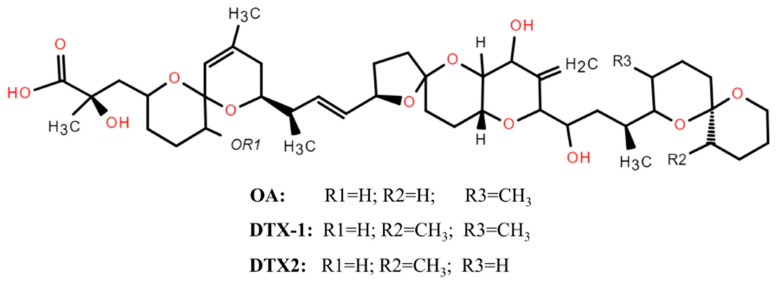
General chemical structure of DSTs, reproduced with permission from the publisher [34].

These lipophilic polyether compounds were originally isolated from the marine sponges *Halichondria okadai* and *H.melanodocia* [14]. They are known to be produced by dinoflagellates of the genera *Dinophysis* and *Prorocentrum*, such as *P. lima*, *P. concavum*, *P. maculosum*, *D. acuminata*, *P. rhathymum*, and *D. fortii* [15]. Structurally, OA and its analogs possess a terminal carboxylic function and a hydroxyl group at C-7. Additionally derivatives named DTX4 to DTX7 have also been identified, particularly in toxin-producing cells (*Dinophysis* spp.) [16]. These toxins share a common polyether carbon backbone, as illustrated in Figure 2, differing mainly in the number and position of methyl groups [17]. These toxins act by inhibiting serine/threonine protein phosphatases (PP1 and specially PP2A), enzymes responsible for catalyzing protein dephosphorylation process [18].

Diarrheic shellfish toxins (DSTs) are found globally with high prevalence, reported in Europe, Japan, and South America [19], with emerging cases documented in Canada, Mexico, India, Thailand, China, and Australia [5]. These toxins accumulate predominantly in soft tissue of bivalves, particularly in the digestive glands [20]. Human exposure occurs primarily through the consumption of contaminated seafood but also via dermal contact or inhalation of aerosolized toxins. Clinical symptoms typically include acute gastrointestinal disturbances such as diarrhea, nausea, vomiting, and abdominal cramps. However, fatalities are rare [21]. DSP outbreaks continue to pose a significant challenge globally, resulting in tens of thousands of intoxication cases annually. These toxins cause ecological disturbances by inducing oxidative stress and immunosuppression in marine organisms [22], with cascading economic consequences due to fishery closures and trade restrictions. DSTs cause both acute gastrointestinal symptoms and long-term cellular, tissue, and intercellular alterations. The acute form manifests rapidly after ingestion, whereas chronic exposure leads to cumulative biochemical and structural damage in exposed organisms.

OA raises serious concerns because of its ability to cause DSP, but also for its broader toxicological effects in humans. These effects emerge as result of chronic exposure to low concentrations through contaminated seafood. OA has been shown to induce various toxic effects, including cytotoxicity [23], neurotoxicity [24], immunotoxicity [25], embryotoxicity [26], genotoxicity [27], and tumor-promoting activity [28]. Understanding the biochemical and molecular basis of OA-group toxin action is essential to link cellular, tissue, and intercellular structural damage with ecological outcomes. This review aims to synthesize the molecular, toxicological, and ecological dimensions of OA-group toxins with emphasis on recent insights into gut microbiota interactions, multi-omics perspectives, and species-specific metabolism. Unlike previous reviews, it integrates mechanistic toxicology with environmental monitoring advances, highlighting emerging research directions for predictive and preventive management.

Several recent reviews have examined OA-group toxins, but most have approached the topic from a limited thematic angle. For instance, Fu et al. [29] provided a comprehensive analysis of OA toxicity, detection methods, and detoxification strategies, yet their review focused mainly on cellular mechanisms and did not explore the broader ecological consequences or the recent developments in monitoring programs. Other reviews available in the literature primarily address either the toxicological pathways of OA [30,31] or the occurrence and regulation of DSP events [32,33], but rarely integrate these dimensions.

Overall, the present review bridges a critical gap by integrating mechanistic toxicology, microbiome-mediated interactions, species-specific metabolic pathways, and advances in environmental monitoring. Unlike previous works that address these elements separately, this synthesis brings them together into a unified and multidisciplinary framework, offering a more predictive and ecologically contextualized understanding of OA-group toxins.

## 2. Mechanisms of Toxicity

OA binds with high affinity to the catalytic subunits of serine/threonine phosphatases (PP1 and PP2A). Structural analyses reveal that the carboxylic acid head region of OA constitutes the principal pharmacophoric domain responsible for its high-affinity binding to serine/threonine protein phosphatases. Structural and crystallographic analyses have shown that this acidic moiety anchors near the catalytic site of PP2A and PP1, forming electrostatic and hydrogen-bond interactions with positively charged residues Tyr265 and Arg89 in PP2A and Tyr272, Arg96, and His125 in PP1 [35,36,37,38,39,40]. This interaction enables OA to occupy the active pocket and block substrate access. Structural modifications around C1–C2 within this head region drastically reduce inhibitory potency, confirming its essential role in enzyme recognition and inhibition [37,38,39,40,41,42]. In contrast, alterations within the hydrophobic tail (C30–C38) exert only minor effects on binding, underscoring the dominant contribution of the carboxylated head to the toxin’s inhibitory activity [37].

## 3. Toxicity and Pathology

DSTs demonstrate distinct oral toxicity profiles, underscoring the importance of using oral exposure models to simulate human intoxication scenarios [43,44]. According to mouse bioassays, dinophysistoxin-1 (DTX1) exhibits the highest toxicity (oral LD_50_ = 487 µg/kg), followed by okadaic acid (OA; 760 µg/kg) and dinophysistoxin-2 (DTX2), which is the least toxic (LD_50_ = 2262 µg/kg). A dose of 1000 µg/kg causes 67% lethality with OA and DTX1, whereas DTX2 requires a threefold higher dose (3000 µg/kg) to induce comparable effects [45]. All oral LD_50_ values reported in this section originate from the same standardized assay type—an acute oral gavage in mice based on the four-level up-and-down procedure [38]. The values for DTX1 (487 µg/kg), OA (760 µg/kg), and DTX2 (2262 µg/kg) were obtained using identical experimental conditions, mouse strains, vehicles, observation periods, and toxin purities. Therefore, the comparisons presented here reflect consistent methodological conditions, despite historical variability in earlier oral toxicity studies. These differences are largely attributed to structural variations in methylation patterns. Specifically, methylation at carbon positions C31 and C35 critically influences gastrointestinal accumulation and systemic toxicity. DTX2, which lacks methylation at C13, exhibits reduced absorption and lower toxicity compared to OA and DTX1, both of which are fully methylated at these positions [43,44]. These modifications affect membrane affinity, intestinal permeability, and bioavailability, leading to greater mucosal retention, particularly within the intestinal epithelium with OA and DTX1 exposure [46].

Following ingestion, DSTs are rapidly absorbed and predominantly distributed to the stomach, small intestine, and large intestine, with only minor hepatic accumulation. Observable clinical symptoms include apathy, piloerection, muscle spasms, dyspnea, and cyanosis, with diarrhea typically occurring within 30 min to 2 h, reflecting swift systemic dissemination [47,48]. Histopathological analysis reveals significant mucosal damage within two hours of exposure [46].

**Figure 3:**
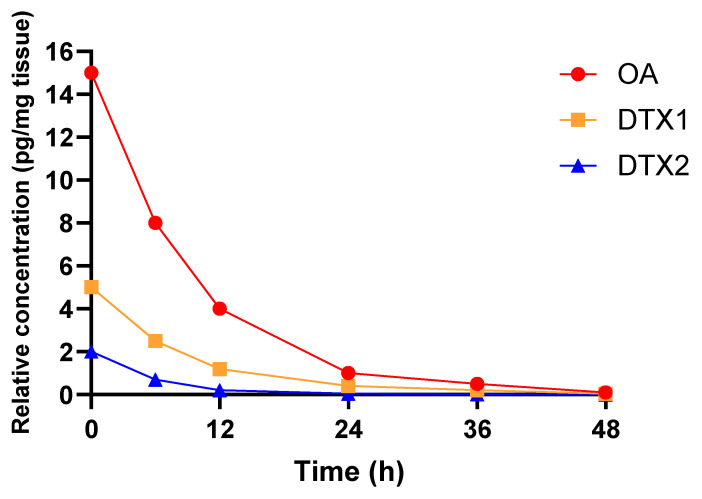
Time-dependent elimination profiles of okadaic acid (OA), dinophysistoxin-1 (DTX1), and dinophysistoxin-2 (DTX2) from 0 to 48 h after oral exposure. Data illustrate the marked differences in absorption and clearance among the three toxins, with OA showing rapid early elimination, DTX1 intermediate kinetics, and DTX2 minimal elimination during the same period. Created with GraphPad Prism 10.

Although normal intestinal peristalsis generally limits toxin retention in specific gut segments, DSTs can disrupt motility, prolong gastrointestinal transit time, and promote enterohepatic recirculation, thereby increasing systemic bioavailability [49,50]. Elimination primarily occurs via feces, though partial urinary excretion has been observed for OA and DTX1 (20–32% recovery within 24 h). In contrast, DTX2 is excreted at significantly lower rates (<2%), unless administered at high doses [44]. The elimination kinetics of OA-group toxins are summarized in Figure 3. OA is rapidly cleared, with major fecal excretion within 2–3 h and 20–32% urinary recovery at 24 h; DTX1 displays similar but slower kinetics and is consistently eliminated in lower amounts, whereas DTX2 shows minimal early excretion, low absorption, and overall recovery below 2% [44,45].

## 4. Cytotoxicity and Gastrointestinal Toxicity

Among its various mechanisms of action, the best characterized involves the inhibition of serine/threonine protein phosphatases (PPs), particularly PP1 and PP2A, as well as PP2B and PP2C [14]. OA is recognized as a potent inhibitor of both PP1 and PP2A, exhibiting a markedly higher binding affinity for PP2A [42]. The cytotoxic effects of OA are primarily manifested through alterations in cell morphology, cytoskeletal disruption, cell cycle arrest, and apoptosis induction [51,52,53]. Damaged cells typically exhibit membrane destabilization, swelling, and metabolic suppression. In various cell types, including neuronal and intestinal models, OA exposure leads to actin filament retraction, microtubule destabilization, and progressive detachment of cells from the substrate [53,54]. Proteomic analyses have revealed the hyperphosphorylation and redistribution of cytoskeleton-regulating proteins such as cofilin-1, plectin, myristoylated alanine-rich c kinase substrate, stathmin, tau, and spectrins [53]. These modifications impair actin polymerization, microtubule organization, and adhesion, activating stress and pro-apoptotic pathways, including caspase-3, extracellular signal-regulated kinases 1 and 2, and c-Fos. This ultimately leads to mitochondrial dysfunction. Elevated levels of 8-hydroxy-2′-deoxyguanosine indicate that oxidative DNA damage also contributes to OA-induced cytotoxicity [52].

In marine bivalves, OA and other DSTs induce multifaceted toxicity involving oxidative stress, immunosuppression, and cytotoxicity. In scallops, exposure to OA leads to an early accumulation of reactive oxygen species (ROS), lipid peroxidation, glutathione depletion, and excessive nitric oxide production. These factors collectively impair antioxidant defenses and disrupt immune gene expression [55]. In *Mytilus galloprovincialis*, exposure to *Prorocentrum lima* triggers tissue-specific and dose-dependent antioxidant responses, including increased glutathione peroxidase and superoxide dismutase (SOD) activity, alongside reduced lipid peroxidation. The differences between gene expression levels and enzymatic activity indicate that post-transcriptional regulatory mechanisms are involved [27].

OA also exhibits significant gastrointestinal toxicity by compromising intestinal barrier integrity. In Caco-2 and T84 human intestinal epithelial cell lines, it decreases transepithelial electrical resistance in a dose-dependent manner, reflecting increased paracellular permeability [56]. This involves the overexpression of *claudin-2*, *claudin-4*, and *zonula occludens-1* and disruption of filamentous actin, all of which weaken epithelial cohesion and barrier function [47]. OA also modulates intestinal physiology at both the molecular and microbial levels. It promotes early inflammatory responses via nuclear factor kappa-light-chain-enhancer of B cell activation and interleukin-8 secretion, even at nanomolar concentrations [54]. Beyond structural disruption, OA alters gut microbiota composition. It increases the abundance of pathogenic genera such as *Escherichia/Shigella*, which negatively correlates with host body weight, while reducing beneficial microbes and promoting gut dysbiosis [45]. In vitro fermentation studies further confirm OA’s dose-dependent impact on the microbiota, showing decreased levels of *Rothia*, *Bacteroides*, and *Helicobacter*, alongside increased abundance of *Faecalitalea* and *Lachnoclostridium* [57]. Notably, *Faecalitalea* abundance is strongly associated with OA-derived metabolites, suggesting a potential role in OA biotransformation and inter-individual variability in toxicity. Multi-omics analyses further demonstrate that OA is metabolically transformed within the gut lumen, most notably into DTX-2, with genera such as *Bacteroides* and *Romboutsia* contributing to these reactions, linking dysbiosis to toxin activation or detoxification pathways [58]. Complementary in vivo evidence from *Galleria mellonella* shows that OA provokes severe midgut epithelial injury, reduced microbiota diversity, and increased susceptibility to secondary *E. coli* infection [59]. Likewise, marine medaka *Oryzias melastigma* exposed to environmentally relevant OA concentrations exhibit *Proteobacteria*-dominated dysbiosis, reduced *Firmicutes*, increased inflammatory markers like C-reactive protein, inducible nitric oxide synthase, and functional shifts associated with carcinogenesis and immune dysregulation [60]. Collectively, these findings support a mechanistic model in which OA-mediated PP2A/PP1 inhibition disrupts epithelial cohesion and mucus integrity, alters microbiota structure and metabolic function, and increases susceptibility to inflammation and secondary infections, thereby amplifying both local and systemic toxicity. Mechanistically, OA-induced dysbiosis further exacerbates toxicity by promoting barrier dysfunction, enhancing luminal inflammation and modifying microbial metabolic pathways involved in toxin bioactivation and detoxification. These microbiota-driven alterations act synergistically with phosphatase inhibition, intensifying intestinal injury and contributing to broader systemic toxic responses [57].

## 5. Neurotoxicity and Embryotoxicity

Although not officially classified as a neurotoxin, OA has demonstrated significant neurotoxic potential, with neuronal toxicity consistently observed at nanomolar concentrations of 20–50 nM in primary cortical neurons and neuroblastoma cells [61]. The nervous system appears particularly sensitive to OA exposure. Early studies reported neuronal cell death associated with tau protein hyperphosphorylation, both in vitro and in vivo [29,34]. Tau is a microtubule-associated protein that loses its stabilizing function when excessively phosphorylated, resulting in microtubule disassembly and neuronal degeneration [62].

Mechanistically, OA induces tau hyperphosphorylation primarily through the inhibition of protein (PP2A), which involves multiple kinases, including extracellular signal-regulated kinases, Jun N-terminal kinases, p38 mitogen-activated protein kinases, protein kinase C, protein kinase A, calcium/calmodulin-dependent kinase II, and calpain [24,63]. These signaling pathways are also connected to oxidative stress, mitochondrial dysfunction, and activation of the apoptotic cascade. In primary cortical neurons, OA induces a strong oxidative response characterized by increased ROS generation, lipid peroxidation, protein carbonylation, and caspase-3/7 activation. These effects are not reversed by estrogens or by mitogen-activated protein kinase/protein kinase inhibition, indicating that broad phosphatase inhibition can override classical neuroprotective pathways and lead to irreversible neuronal injury [61].

**Figure 4:**
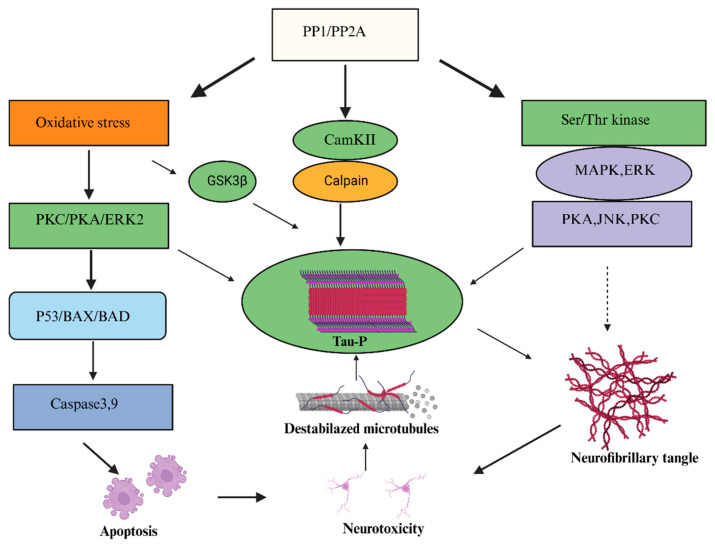
This representative diagram depicts the overall effect of OKA on oxidative stress, regulation of kinase activity, apoptosis of tau hyperphosphorylation, and neurofibrillary tangle formation. This diagram was created de novo by the authors using Biorender.com. Abbreviations: OA, okadaic acid; Tau-P, phosphorylated tau protein; ROS, reactive oxygen species; ERK, extracellular signal-regulated kinase; JNK, c-Jun N-terminal kinase; PP2A, protein phosphatase 2A; ATP, adenosine triphosphate; MAPK, mitogen-activated protein kinase.

Together, these factors contribute to neurotoxicity and Alzheimer’s disease-like pathology, which include β-amyloid accumulation and neurofibrillary tangle formation [64]. Figure 4 summarizes this cascade, illustrating how PP2A inhibition and kinase hyperactivation converge on tau phosphorylation, microtubule destabilization, and apoptotic neuronal loss. In rodents, intracerebral administration of OA produces hippocampal damage, impaired spatial memory, and tau/β-amyloid pathology consistent with Alzheimer’s disease models [65,66,67]. More recent investigations have broadened the mechanistic understanding of OA-induced neurotoxicity. In SH-SY5Y neurons, OA impairs cholinergic neurotransmission by reducing α7- and β2-containing nicotinic acetylcholine receptor activity, indicating early synaptic dysfunction independent of glutamatergic pathways [68]. In vivo, OA administration rapidly induces widespread tau phosphorylation that extends from the injection site to distant cortical and hippocampal regions followed by the accumulation of insoluble and thioflavin-positive tau aggregates, indicating that OA can initiate tau pathology across connected neuronal circuits [69].

Transcriptomic analyses in *Oryzias melastigma* further reveal that OA disrupts key neuronal pathways, notably synaptic vesicle cycling, glutamatergic signaling, and long-term potentiation, including downregulation of alpha-amino-3-methyl-5-phosphonopropionic acid and N-methyl-D-aspartate receptor subunits. These molecular alterations impair synaptic transmission and reduce locomotor performance. OA also upregulates neuronal nitric oxide synthase and interferes with NO-dependent neuromodulatory pathways while concurrently dysregulating DNA repair and cell cycle processes (nucleotide excision repair, mismatch repair, p53 signaling), indicating a combined neurotoxic and genotoxic stress response [67]. Additional evidence shows alterations in axon guidance, gap-junction communication, and apoptosis-related pathways in okadaic acid-exposed marine medaka [70].

Beyond neurotoxicity, OA also exhibits marked embryotoxic and neurodevelopmental effects across various vertebrate species, including *Xenopus laevis*, *Oryzias latipes*, and *Gallus gallus*. OA delays embryonic development and increases the incidence of malformations and mortality [71,72,73]. OA can cross the placental barrier in mice and has been shown to accumulate at higher concentrations in fetal tissues than in maternal tissues, indicating enhanced fetal susceptibility [74,75]. In avian models, OA induces neural tube defects and craniofacial malformations. OA disrupts the signaling pathways of bone morphogenetic protein 4 and Sonic Hedgehog and impairs neural crest cell migration via inhibition of PP2A [49].

## 6. Genotoxicity and Immunotoxicity

OA is a potent genotoxic and tumor-promoting compound. It causes DNA double-strand breaks, disrupts chromatin organization, and impairs DNA repair mechanisms through inhibition of protein phosphatases PP1 and PP2A. This results in the dysregulation of the p53 pathway and abnormal histone modifications [48,67]. These alterations contribute to genomic instability, apoptosis, and uncontrolled cell proliferation.

**Figure 5:**
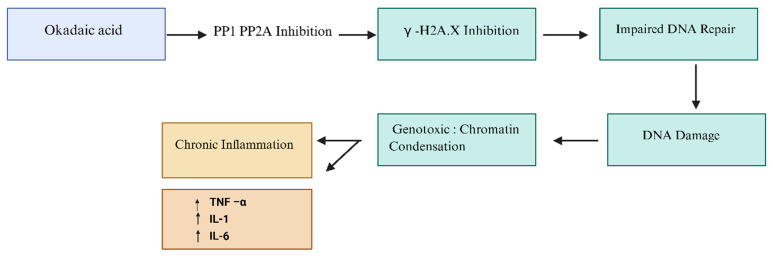
Representative diagram illustrating the genotoxic pathways activated by OA. OA inhibits protein phosphatases PP1 and PP2A, leading to reduced γ-H2A.X activation and impaired DNA repair, which ultimately results in DNA damage and chromatin condensation. OA also triggers the release of pro-inflammatory cytokines (TNF-α, IL-1, IL-6), promoting chronic inflammation and contributing to long-term cellular stress. Created with BioRender.com. Abbreviations: OA, okadaic acid; PP1, protein phosphatase 1; PP2A, protein phosphatase 2A; γ-H2A.X, phosphorylated H2A histone family member X; TNF-α, tumor necrosis factor alpha; IL-1, interleukin-1; IL-6, interleukin-6.

In vivo evidence substantiates OA’s genotoxic effects, as demonstrated by angiogenesis defects during embryonic development [52] and DNA integrity disruption in multiple model organisms [30]. As shown in Figure 5, PP1/PP2A inhibition impairs the DNA damage response by blocking phosphorylated histone family member X activation, leading to defective DNA repair, chromatin condensation, and accumulation of DNA damage. These events contribute to the cytotoxicity of OA, including its genotoxic effects. Simultaneously, OA stimulates the production of pro-inflammatory cytokines such as TNF-α, IL-1, and IL-6, promoting chronic inflammation, which represents a key mechanism underlying its long-term toxicity.

In vivo studies also highlight the immunotoxic effects of OA. In Swiss mice fed OA-contaminated mussels, systemic immune alterations were observed, characterized by initial immune stimulation followed by immunosuppression [55,76]. In ex vivo porcine intestinal explants, exposure to OA rapidly led to the formation of lamellar-body organelles, associated with lysosomal activity. This suggests early immune or stress-related responses at the cellular level [77].

## 7. Metabolism and Esterification of DST in Bivalves

The elimination of DSTs in bivalves primarily occurs through esterification with fatty acids, resulting in the formation of 7-O-acyl derivatives, known as DTX3 esters (detoxification metabolites). This detoxification pathway found in *Mytilus edulis*, *M. galloprovincialis*, *Crassostrea virginica*, and *Ilyanassa obsoleta* facilitates toxin excretion through feces while limiting the release of free toxins into the surrounding seawater [78,79,80,81,82]. In mussels, DTX2 displays slower depuration due to reduced esterification rates, and fatty acids such as palmitic acid (C16:0), palmitoleic acid (C16:1), stearic acid (C18:0), oleic acid (C18:1), eicosenoic acid (C20:1), and arachidic acid (C20:0) are commonly involved in conjugation process [83].

Toxin accumulation is often associated with a reduction in polyunsaturated fatty acids, particularly eicosapentaenoic acid and docosahexaenoic acid. This decline leads to a lower nutritional quality of bivalves during depuration. This process is accompanied by the upregulation of key lipid metabolism enzymes, including acetyl-CoA carboxylase, fatty acid synthase, lipoprotein lipase, and hepatic lipase HL. This upregulation occurs primarily in the digestive gland, which is the main site of toxin storage [84]. Biochemical studies have confirmed that the acyl-CoA:OA acyltransferase is active in the microsomal and mitochondrial fractions of the digestive gland and strongly correlates with cytochrome c reductase activity, indicating that the endoplasmic reticulum is the principal site of OA esterification [81]. OA may undergo hydrolysis and reconjugation, forming DTX3 from diol-esters such as DTX4, DTX5, and DTX6 [85].

Esterification efficiency varies by species and toxin. Certain species, such as *Cerastoderma edule*, *Scrobicularia plana*, *Venerupis pullastra*, *Crassostrea gigas*, and *Ensis* spp., can rapidly achieve near-complete (100%) esterification. In contrast, mussels like *M. galloprovincialis*, *M. coruscus*, and *C. grayanus* generally exhibit lower esterification rates [86,87,88]. In most commercial bivalves, OA and DTX2 are extensively esterified, but species of *Mytilus* consistently show lower proportions of esterified derivatives [89]. More than 90% of total DSTs in several invertebrates occur as fatty acid esters, demonstrating that esterification is the dominant metabolic pathway for OA-group toxins [90]. Hybrid diol–fatty-acid esters have also been identified in mussels, confirming that multiple esterified forms are produced through active metabolic processes in the digestive gland [91].

These metabolic differences result in strong bivalve species-specific variability in toxin retention. Cockles (*Cerastoderma edule*) display a rapid depuration rate for both OA and DTX2, eliminating toxins efficiently from the digestive gland, the gills, the intestine, and other soft tissues, whereas mussels (*Mytilus galloprovincialis*) depurate toxins much more slowly, particularly DTX2, which is lost at nearly half the rate of OA [78]. As illustrated in Table 1, *Mytilus* species tend to act as long-lasting toxin reservoirs, while cockles and clams depurate toxins more rapidly and therefore pose a shorter and less persistent ecological risk during DSP events.

OA uptake in bivalves may occur via phagocytosis (when associated with *Dinophysis* debris), pinocytosis, or passive diffusion. Once internalized, the toxins are processed through endosomal–lysosomal pathways [92]. At the molecular level, exposure to OA induces the overexpression of ATP-binding cassette (ABC) transporters in mussels, including multidrug resistance protein 1 (MDR1;P-glycoprotein) and multidrug resistance protein 2 (MRP2), which are involved in xenobiotic efflux and cholesterol homeostasis [67,93]. In humans, OA is predominantly metabolized by cytochrome enzymes of the cytochrome P450 family during phase I biotransformation. These NADPH-dependent processes generate hydroxylated and oxidized metabolites. Significant interspecies differences in metabolism are noted. While rat liver enzymes convert OA into less cytotoxic derivatives, human enzymes may generate more reactive and toxic intermediates, thereby potentially exacerbating OA-induced cellular toxicity [62].

**Table 1:** Summary of ecotoxicological effects of OA-group toxins in species. Abbreviations: NR, not reported.

Species	Toxin Concentration	Exposure Route	Endpoint(s)	References
*Mytilus galloprovincialis*	0.5–0.54 mg OA/g Organ/matrix: hepatopancreas (HP)	Oral uptake via contaminated microalgae/purified OA	Oxidative stress (↑ ROS), reduced SOD/catalase activity, lysosomal destabilization, immune suppression (hemocytes), impaired cellular homeostasis	[94]
*Cerastoderma edule* (cockle)	NR Organ/matrix: whole soft tissues	OA-contaminated seston ingestion	Rapid depuration; high esterification efficiency (>98% acyl derivatives)	[89,90]
*Crassostrea gigas*	3 µg OA eq/mL Organ/matrix: hemocytes (in vitro exposure)	In vitro exposure (hemocytes) to OA/DTX	Programmed cell death (apoptosis/pyroptosis), caspase-1 and caspase-7 modulation, low cytotoxicity, high esterification efficiency, potential metabolic alterations	[56,67]
*Scrobicularia plana*	2.1–1780 ng/L OA Organ/matrix: environmental water (water column)	Exposure to OA in the environment	Efficient esterification; limited accumulation	[88,89]
*Patinopecten yessoensis*	NR Organ/matrix: whole soft tissues	Natural exposure to Dinophysis toxins	Biotransformation of DTX-1 → DTX-3 (esterification)	[89]
*Ilyanassa obsoleta*	NR Organ/matrix: whole soft tissues	Exposure to environmental OA	Esterification capacity known; detoxification via DTX3	[95]

Note: Reported toxin concentrations may refer to either (i) accumulated levels measured in specific tissues (e.g., hepatopancreas, whole soft tissues, hemocytes) or (ii) environmental exposure concentrations (e.g., water column). The relevant matrix is specified for each species in the “Organ/matrix” column. Symbols → indicate biotransformation and ↑ indicates an increase

## 8. Ecotoxicological Effects of DSTs on Aquatic Organisms

Natural high-density proliferations of toxin-producing microorganisms occur in both marine and freshwater environments and have substantial socioeconomic and ecological consequences, mainly due to the contamination of water and seafood. Exposure to OA and DTXs in fish has adverse effects across multiple developmental stages. Species such as the longfin yellowtail (*Seriola rivoliana*) and zebrafish (*Danio rerio*) exhibit reduced hatching success and developmental delays in the early stages of their lives, probably related to the inhibition of serine/threonine phosphatases [26]. Species such as European seabass (*Dicentrarchus labrax*), gilthead seabream (*Sparus aurata*), and zebra seabream (*Diplodus cervinus*) display signs of oxidative stress and histopathological lesions in the liver and gills at both juvenile and adult stages. Behavioral alterations such as impaired swimming performance and reduced feeding activity have also been documented, with mortality observed under severe exposure conditions [22,96].

Comparative studies indicate that dietary exposure generally results in higher toxin accumulation and more pronounced physiological impairments than waterborne exposure, underscoring trophic transfer as a critical route for OA-group toxin impact in fish populations [96]. Although fish tend to accumulate free OA in the viscera over short periods, the ecological risks posed by these toxins may be exacerbated under environmental stress conditions. This underscores the importance of conducting further studies at environmentally relevant concentrations [96]. The oxidative stress response induced by OA exposure is reflected by increased activity of antioxidant enzymes such as catalase and glutathione along with elevated levels of malondialdehyde, a marker of lipid peroxidation in the liver within 24 h post-exposure, confirming the generation of ROS [97].

While fish represent higher trophic levels that reflect the bioaccumulative and physiological impacts of DSTs, bivalves act as primary vectors and biological reservoirs, exhibiting adaptive responses that modulate toxin transfer within marine food webs. In addition to their effects on fish, DSTs are predominantly accumulated by filter-feeding organisms including bivalves [34]. However, recent surveys indicate that these toxins also accumulate in invertebrates. Gastropods such as *Patella* spp. and *Onchidella celtica* consistently contained OA in natural populations across Madeira, the Azores, and Morocco [98]. Echinoderms, particularly *Paracentrotus lividus*, represent additional vectors with measurable OA levels in field samples. Experimental studies further demonstrate pronounced behavioral and physiological sensitivity to OA in sea urchins such as *Strongylocentrotus intermedius* [99]. Consistent with these observations, large-scale *Dinophysis* blooms have revealed OA contamination in a broader range of benthic taxa, including crustaceans such as *Callichirus major* and echinoderms like *Mellita quinquiesperforata* exposed through grazing on microalgal films, sediment-associated biofilms, and organic deposits enriched with residual OA and its analogs, highlighting additional species that can act as previously unrecognized vectors [100]. Zooplankton also contributes to benthic exposure by releasing OA-containing fecal pellets that sink and are consumed by detritivores [101]. In addition crustaceans such as *Carcinus maenas* and *Cancer pagurus* can also accumulate OA esters through predation on contaminated bivalves [102,103], while benthic annelids such as *Enchytraeus crypticus* retain OA from sediment-associated material [104], reinforcing the multi-species circulation of DSTs within benthic food webs. These findings indicate that DSTs circulate through a broader range of benthic invertebrates than previously recognized. Although bivalves can tolerate high intracellular concentrations of DSTs without immediate lethality, they exhibit a range of physiological and cellular responses, including valve closure, reduced filtration rates, and histopathological changes, oxidative stress, and immune modulation [105].

To mitigate toxin-induced damage, bivalves activate a suite of detoxification and antioxidant defense mechanisms. These include phase I and II enzymes such as cytochrome P450 and glutathione S-transferases, ATP-binding cassette (ABC) transporters, and activation of the nuclear factor erythroid 2-related factor 2 signaling pathway, which collectively enhance xenobiotic clearance and redox homeostasis [65,66]. Transcriptomic and proteomic studies provide deeper evidence that ABC transporters play a central role in DST detoxification across aquatic taxa. In bivalves, exposure to Prorocentrum lima or purified OA induces strong modulation of multiple ABC family members, including ABCB10, ABCC1, ABCC5, and P-glycoprotein, which increase in expression proportionally with toxin accumulation in gill and digestive gland tissues. In fish, OA alters hepatic expression of ABCA3 and ABCA5, indicating that ABC-mediated transport also contributes to toxin handling in vertebrates [106]. These adaptive responses enable bivalves to survive in toxin-rich environments and facilitate the bioaccumulation and ecological cycling of DSTs posing a significant risk to seafood safety and human health. Importantly, the ecological risks associated with OA-group toxins may be underestimated, as environmental stressors can exacerbate toxic effects, highlighting the need for studies conducted at environmentally relevant toxin concentrations.

## 9. Effects of DSTs on Marine Mammals

The toxic effects of OA on marine mammals remain poorly understood due to the paucity of toxicological data, despite documented environmental exposure. OA was detected at low concentrations in bottlenose dolphins (*Tursiops truncatus*) stranded during a 2008 mortality event in Texas, coinciding with blooms of *Dinophysis* and *Prorocentrum* species, known to produce DSTs [107]. Additional evidence of OA exposure has been reported in healthy individuals of South American sea lions (*Otaria byronia*) and Peruvian fur seals (*Arctocephalus australis*) in coastal Peru, where OA was detected in fecal samples from approximately 33% of tested individuals at concentrations ranging from 0.5 to 36 ng/g [108]. Consistent with these findings, recent observations in large-scale *Dinophysis* blooms have revealed OA accumulation in the livers of Guiana dolphins (*Sotalia guianensis*) and in stranded Magellanic penguins (*Spheniscus magellanicus*), both of which exhibited gastrointestinal and hepatic lesions, suggesting potential sublethal or chronic impacts associated with DSP toxin exposure [100]. These findings suggest that marine mammals may accumulate DSTs through trophic transfer in highly productive upwelling systems such as the Humboldt Current. While acute toxicity has not been clearly demonstrated, the potential for chronic or sublethal effects remains insufficiently characterized and warrants further investigation.

## 10. Detection Techniques

The detection and quantification of DSTs, including OA and its analogs, are critical for ensuring seafood safety and monitoring (HAB). A range of analytical techniques are available, each with specific advantages and limitations depending on the context of use. Table 2 summarizes the key methods used for DST detection, including chromatographic, immunological, and bioanalytical approaches along with their advantages and disadvantages, and the corresponding limits of detection (LOD) and quantification (LOQ) when available.

## 11. Perspectives

Future research should prioritize longitudinal investigations to evaluate the chronic and sublethal effects of OA on both human consumers and marine wildlife. This should be complemented by comparative molecular studies aimed at elucidating the metabolism and detoxification mechanisms of the toxin across different bivalve and fish species. Efforts are also needed to develop advanced analytical methodologies capable of detecting the full spectrum of DST forms, including free, esterified, and conjugated derivatives, in both environmental and biological matrices. Furthermore, the assessment of combined exposures to multiple toxins and other environmental contaminants is essential to better approximate real-world scenarios and support the establishment of more comprehensive seafood safety regulations. Investigations into the molecular basis of inter-individual and inter-specific differences in sensitivity employing integrative omics approaches, such as genomics, transcriptomics, and metabolomics, will provide deeper insight into susceptibility mechanisms. Multi-omics approaches offer valuable opportunities to advance our understanding of chronic exposure and species-specific responses to DSTs. Transcriptomics can reveal early gene expression changes associated with sublethal exposure, while proteomics can identify alterations in stress response pathways and post-translational regulation. Metabolomics provide insight into shifts in energy metabolism and detoxification processes. Integrating these omics layers across bivalve species would improve predictions of susceptibility, detoxification efficiency, and long-term ecological risk. Artificial intelligence and machine learning frameworks offer new opportunities to forecast HAB toxicity and support real-time decisions. When combined with metabolomics and predictive modeling, these tools can improve the early detection of subtle metabolic shifts associated with chronic low-dose exposures. Finally, strengthening policy frameworks is especially critical for developing countries, where limited analytical infrastructure and irregular monitoring efforts heighten the risk of undetected DSP contamination. Implementing cost-effective biosensor networks and standardized reporting protocols would help reduce these vulnerabilities.

## 12. Conclusions

DSTs represent one of the most challenging groups of phycotoxins due to their ability to affect multiple biological systems simultaneously and their wide ecological footprint. Beyond their well-recognized phosphatase-inhibitory activity, accumulating evidence reveals that OA-group toxins influence a diverse array of molecular networks involved in cell signaling, energy regulation, developmental processes, and organismal homeostasis. Their multilayered effects indicate that the consequences of exposure extend far beyond acute gastrointestinal symptoms and may contribute to long-term physiological and ecological disturbances that remain insufficiently understood. Despite progress in analytical detection and structural characterization of OA analogs, substantial uncertainties persist regarding the cumulative impacts of recurrent low-dose exposures, species-specific metabolic transformations, and co-occurrence with other HAB-related toxins under realistic field conditions. Addressing these knowledge gaps is essential to develop reliable early-warning tools and to refine the toxicological thresholds used in shellfish safety programs. Overall, this review integrates molecular, toxicological, and ecological evidence to provide a comprehensive understanding of OA-group toxin actions across biological scales. Addressing the remaining knowledge gaps, particularly those related to chronic exposure, inter-species variability, and ecosystem-level consequences, will be essential for refining risk assessment frameworks and safeguarding marine ecosystems and human health in the context of increasingly frequent and intense HAB events.

## Figures and Tables

**Table 2:** Advantages and disadvantages of different detection methods. Abbreviations: fg, femtogram; pg, picogram; ng, nanogram; L, liter, HP, hepatopancreas, ND: not detected.

Techniques	Advantages	Disadvantages	Limit of Detection LOD	Limit of Quantification LOQ	References
LC-MS/MS (Liquid Chromatography–Tandem Mass Spectrometry)	High sensitivity and specificity; accurate quantification of OA and analogs; standard in regulatory monitoring programs; applicable to various matrices	Requires expensive instrumentation and skilled personnel; non-portable; complex sample pretreatment often needed.	0.045 mg/g HP (OA)/5–10 ng/g (tissues)/0.2 ng/mL	0.135 mg/g HP (OA)/1.3 ng/mL	[87]
HRMS (High-Resolution Mass Spectrometry)	Accurate mass determination analysis; enables untargeted screening and identification of novel analogs; valuable in research and environmental surveys	High costs; complex data analysis; less suitable for routine large-scale monitoring	5 fg (column); 0.4 ng/L (particulate); 0.3 ng/L (seawater)	15 fg (column); 1 ng/L (particulate/seawater)	[4,109]
Biosensors/Immunosensors/SPR	Rapid and on-site detection; field-deployable; cost-effective; adaptable detection modalities, allows diverse detection modalities (optical, electrochemical, luminescent)	Lower sensitivity compared to LC-MS/MS; potential cross-reactivity; requires validation for complex samples; may suffer from environmental stability	2.6 ng/mL (=2.6 µg/L)	ND	[110,111]
ELISA (Enzyme-Linked Immunosorbent Assay)	Cost-effective and sensitive; enables high-throughput screening; simple execution	Cannot differentiate toxin analogs; potential false positives; results dependent on antibody specificity	12 pg/mL	ND	[112,113]
RIA (Radioimmunoassay)	High sensitivity; suitable for quantitative analysis of toxins	Uses radioactive materials with regulatory and disposal constraints decreasing use due to safety concerns.	~1 ng/mL	ND	[114]
MBA (Mouse Bioassay)	Historically reliable; indicates overall toxicity profile without complex equipment	Low sensitivity and reproducibility; ethical concerns; unable to distinguish toxin types; time-consuming	20 µg OA/kg	ND	[115]
HPLC (High-Performance Liquid Chromatography)	Precise quantification; suitable for routine monitoring; highly accurate and applicable for routine monitoring; requires only small sample volumes	Requires toxin standard; limited multiplexing; no specific detectors; costly instrumentation	0.015 mg/g HP	0.015 mg/g HP	[116,117]
TLC (Thin-Layer Chromatography)	Simple; low-cost; no need for advanced method; useful for qualitative analysis	Low sensitivity and specificity; not suitable for quantification; labor-intensive and prone to subjective interpretation	ND	ND	[118]
MEKC (Micellar Electrokinetic Chromatography)	Fast and efficient separation; low sample and reagent consumption; can separate neutral and charged analytes	Sensitive to variations in buffer composition and temperature; limited handling of complex biological samples	40 pg (on-column); ~10 ng/g in mussel tissue	ND	[119,120]
GC (Gas Chromatography)	High sensitivity and accuracy for volatile and derivatized toxins; robust analytical method	Requires derivatization for non-volatile toxins; high cost; complex data interpretation; potential false positives if preparation is inadequate	~50 ng (after derivatization; low sensitivity)	ND	[119]

## Data Availability

The data presented in this study are available on request from the corresponding author.
